# Supplementary material for: Trimethylamine N-oxide impairs β-cell function and glucose tolerance
Source: Nat Commun. 2024 Mar 21;15:2526. doi: 10.1038/s41467-024-46829-0 (PMC10957989; doi:10.1038/s41467-024-46829-0)
Supplement: Supplementary file 1 — Supplementary Information [file 41467_2024_46829_MOESM1_ESM.pdf]

Trimethylamine N-oxide impairs β-cell function and glucose tolerance

Lijuan Kong[1,2,3,#], Qijin Zhao[1,2,3,#], Xiaojing Jiang[1,2,3,#], Jinping Hu[1], Qian Jiang[1,2,3], Li Sheng[1], Xiaohong Peng[4,5], Shusen Wang[6], Yibing Chen[1,2,3], Yanjun Wan[1,2,3], Shaocong Hou[1,2,3], Xingfeng Liu[1,2,3], Chunxiao Ma[1,2,3], Yan Li[1], Li Quan[5], Liangyi Chen[4,5], Bing Cui[1,3], Pingping Li[1,2,3✉]

Supplementary Figures and Legends

Supplementary Tables

Supplementary Figures and Legends

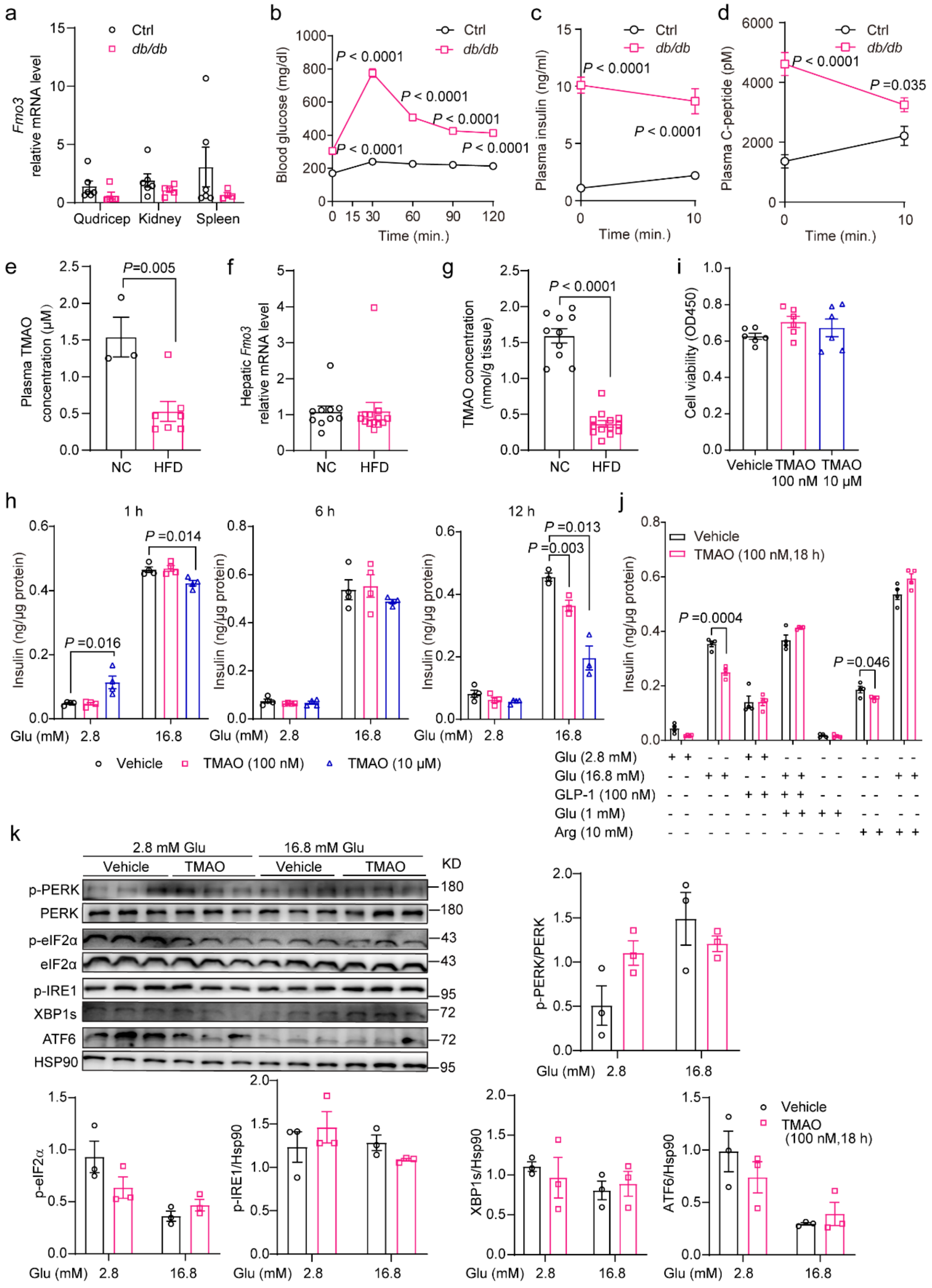

Supplementary Figure 1. TMAO time-dependently inhibited GSIS, related to Figure 1. a *Fmo3* mRNA levels in different tissues of 15-week-old male control (n=6

mice) and *db/db* mice (n=5 mice). **b** Oral glucose tolerance test of 9-week-old male control and *db/db* mice. n=12 mice. **c** Plasma insulin during the glucose tolerance test. n=8 (control), or 11 (*db/db*) mice. **d** Plasma C-peptide during the glucose tolerance test. n=10 (control), or 11 (*db/db*) mice. **e** Plasma TMAO concentration in male NC (n=3 mice) and HFD-fed (22 weeks of feeding) mice (n=7 mice). **f** Hepatic *Fmo3* mRNA levels in male NC (n=10 mice) and HFD-fed (19 weeks of feeding) mice (n=13 mice). **g** Pancreatic TMAO concentration in male NC (n=10 mice) and HFD-fed (19 weeks of feeding) mice (n=10 mice). **h** GSIS of MIN6 cells treated with or without TMAO for 1, 6, and 12 h. n=3 (12 h at 16.8 mM Glu), or 4 (others) biologically independent cell samples. **i** CCK-8 assay of MIN6 cells treated with TMAO (100 nM and 10 μM) for 18 h. n=6 biologically independent cell samples. **j** Glucose, GLP-1 and arginine stimulated insulin secretion of MIN6 cells treated with or without TMAO (100 nM, 18 h). n=4 biologically independent cell samples. **k** ER stress-related protein levels in MIN6 cells treated with or without TMAO (100 nM, 18 h). n=3 biologically independent cell samples. Statistical significance was calculated (**b-h, j**) by two-sided Student's t-test. The data are presented as mean ± SEM. Arg, arginine; GLP-1, glucagon-like peptide-1. Source data are provided as a Source Data file.

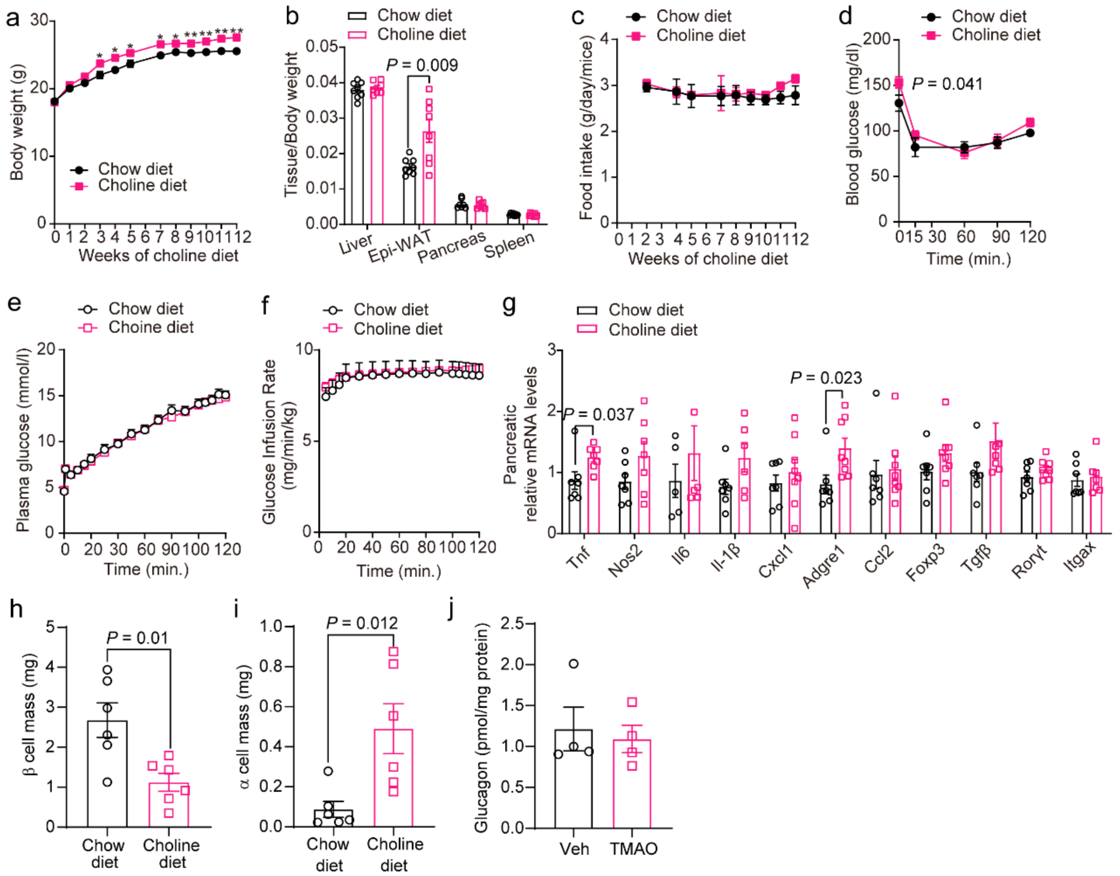

Supplementary Figure 2. Elevated inflammation in islets of mice fed choline, related to Figure 2. a Body weight during choline diet feeding in male C57BL/6J mice. n=15 mice. **b** Tissue weight to body weight ratio in male chow- and choline diet-fed (13 weeks) mice. n=8 mice. **c** Food intake during choline diet feeding in male C57BL/6J mice. n=3 cages. **d** ITT of male chow- (n=12 mice) and choline diet-fed (10 weeks) mice (n=14 mice). The insulin dose was 0.3 U/kg of body weight. **e-f** Plasma glucose (**e**) and GIR (**f**) during hyperglycemic clamp of male chow- (n=7 mice) and choline diet-fed (13 weeks) mice (n=9 mice). **g** Pancreatic inflammatory gene mRNA levels in male chow- and choline diet-fed (13 weeks) mice. n=7 mice. **h-i** Quantification of β- and α- cell mass in male chow- and choline diet-fed (13 weeks) mice. n=6 mice. **j** Glucagon secretion under low glucose conditions in α cell line αTC1-6 cells treated with or without TMAO (100 nM, 18 h). n=4 biologically independent cell samples. Statistical significance was calculated (**a-b, d, g-i**) by two-sided Student's t-test. The data are presented as mean ± SEM. *P* values in **a** denoted by asterisks (from left to

right): $P = 0.009$, $P = 0.005$, $P = 0.014$, $P = 0.011$, $P = 0.033$, $P = 0.023$, $P = 0.01$, $P = 0.003$, $P = 0.001$. Mice: C57BL/6J, choline diet feeding from 8 weeks old (**a-i**). Source data are provided as a Source Data file.

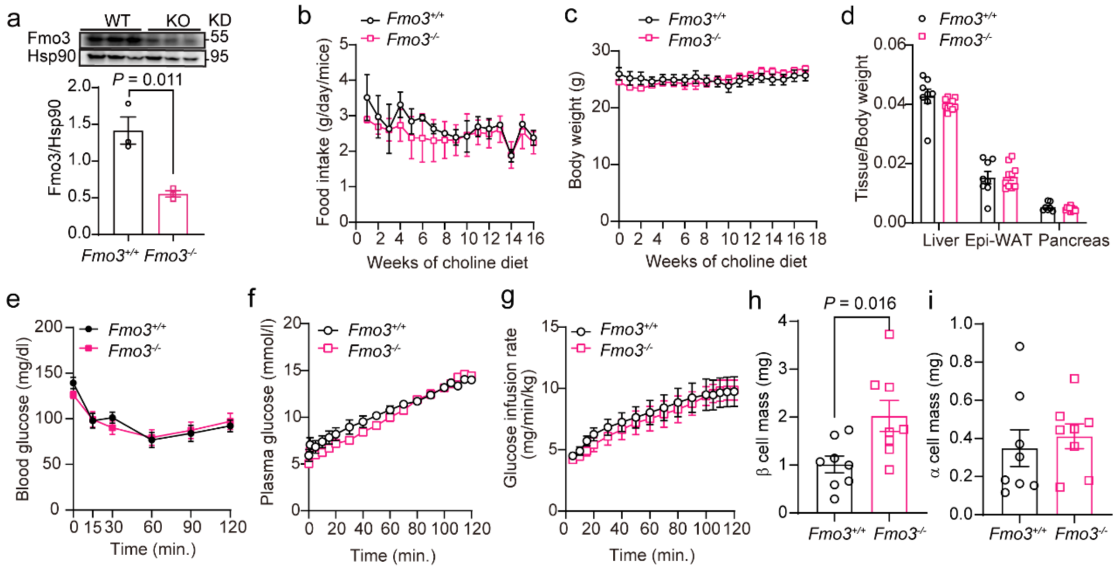

Supplementary Figure 3. Similar insulin sensitivity in control and Fmo3 KO mice, related to Figure 3. a Hepatic Fmo3 protein levels of male *Fmo3*$^{+/+}$ and *Fmo3*$^{-/-}$ mice. n=3 mice. **b** Food intake of male *Fmo3*$^{+/+}$ and *Fmo3*$^{-/-}$ mice during choline diet feeding. n=2 cages. **c** Body weight of male *Fmo3*$^{+/+}$ (n=8 mice) and *Fmo3*$^{-/-}$ mice (n=10 mice) during choline diet feeding. **d** The ratio of tissue weight to body weight in male choline diet-fed *Fmo3*$^{+/+}$ (n=8 mice) and *Fmo3*$^{-/-}$ mice (n=10 mice). **e** ITTs of male choline diet-fed (16 weeks) *Fmo3*$^{+/+}$ (n=7 mice) and *Fmo3*$^{-/-}$ mice (n=9 mice). **f-g** Plasma glucose (**f**) and GIR (**g**) during hyperglycemic clamp in male choline diet-fed (18 weeks) *Fmo3*$^{+/+}$ and *Fmo3*$^{-/-}$ mice. n=5 mice. **h-i** Quantification of β- and α- cell mass in male choline diet-fed (18 weeks) *Fmo3*$^{+/+}$ and *Fmo3*$^{-/-}$ mice. n=8 mice. Statistical significance was calculated (**a, h**) by two-sided Student's t-test. The data are presented as mean ± SEM. Mice: C57BL/6J, choline diet feeding from 8 weeks old (**a-i**). Source data are provided as a Source Data file.

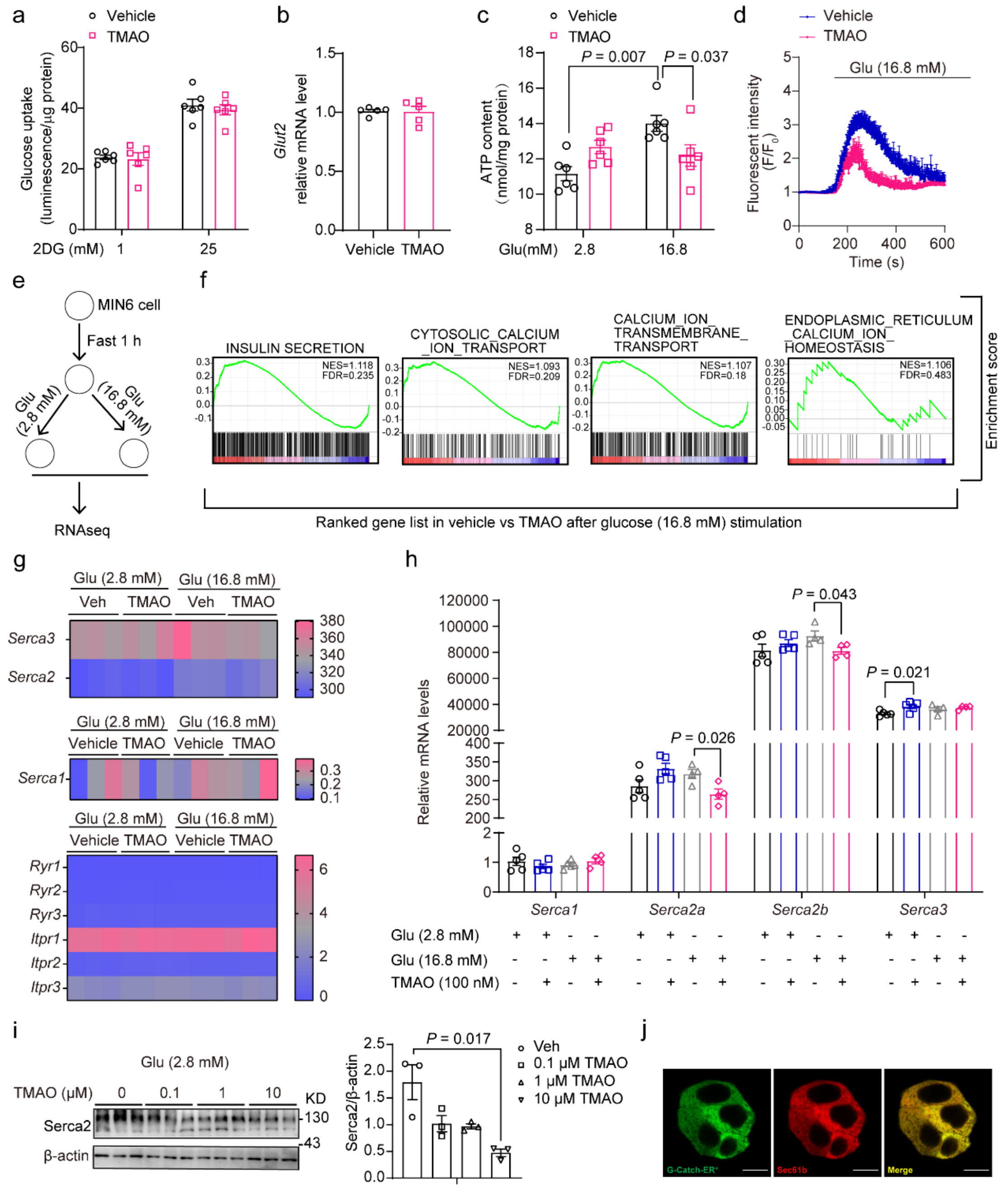

Supplementary Figure 4. TMAO did not alter glucose uptake, related to Figure 4. **a** Glucose uptake of MIN6 cells treated with or without TMAO (100 nM, 18 h). n=6 biologically independent cell samples. **b** *Glut2* mRNA levels in MIN6 cells treated with or without TMAO (1 μM, 18 h). n=5 biologically independent cell samples. **c** ATP content in male mouse primary islets treated with or without TMAO (1 μM, 18 h). n=6 biologically independent samples. **d** Cytosolic calcium dynamics with GCaMP6f stimulated by high glucose (16.8 mM) in MIN6 cells treated with or without TMAO (100 nM, 18 h). n=13 (vehicle), or 14 (TMAO) cells from 6 samples. **e** The treatment

process of MIN6 cells for RNA-seq. **f** GSEA plot of insulin secretion, cytosolic calcium ion transport, calcium ion transmembrane transport and ER calcium ion homeostasis biological processes in vehicle- and TMAO (100 nM, 18 h)-treated MIN6 cells after high-glucose (16.8 mM) stimulation. Cytosolic calcium ion transport-, calcium ion transmembrane transport- and ER calcium ion homeostasis-related genes were included in insulin secretion-related genes. **g** Heatmap of *Serca2, Serca3, Serca1, Ryr1, Ryr2, Ryr3, Itpr1, Itpr2,* and *Itpr3* mRNA levels in MIN6 cells treated with or without TMAO (100 nM, 18 h) by RNA-seq. n=3 biologically independent cell samples. The heatmap was generated using fragments per kilobase per million (FPKM) values. **h** *Serca1, Serca2a, Serca2b and Serca3* mRNA levels in MIN6 cells treated with or without TMAO (100 nM, 18 h). n=5 biologically independent cell samples. **i** Serca2 protein levels in MIN6 cells treated with or without TMAO (100 nM, 1 μM or 10 μM, 18 h) under 2.8 mM glucose conditions. n=3 biologically independent cell samples. **j** Spatial localization of ER calcium indicator G-Catch-ER+ and ER marker Sec61β in MIN6 cells. Scale bar: 10 μm. Statistical significance was calculated (**c, h-i**) by two-sided Student's t-test. The data are presented as mean ± SEM. Mice: C57BL/6J, 15 weeks old (**c**). Source data are provided as a Source Data file.

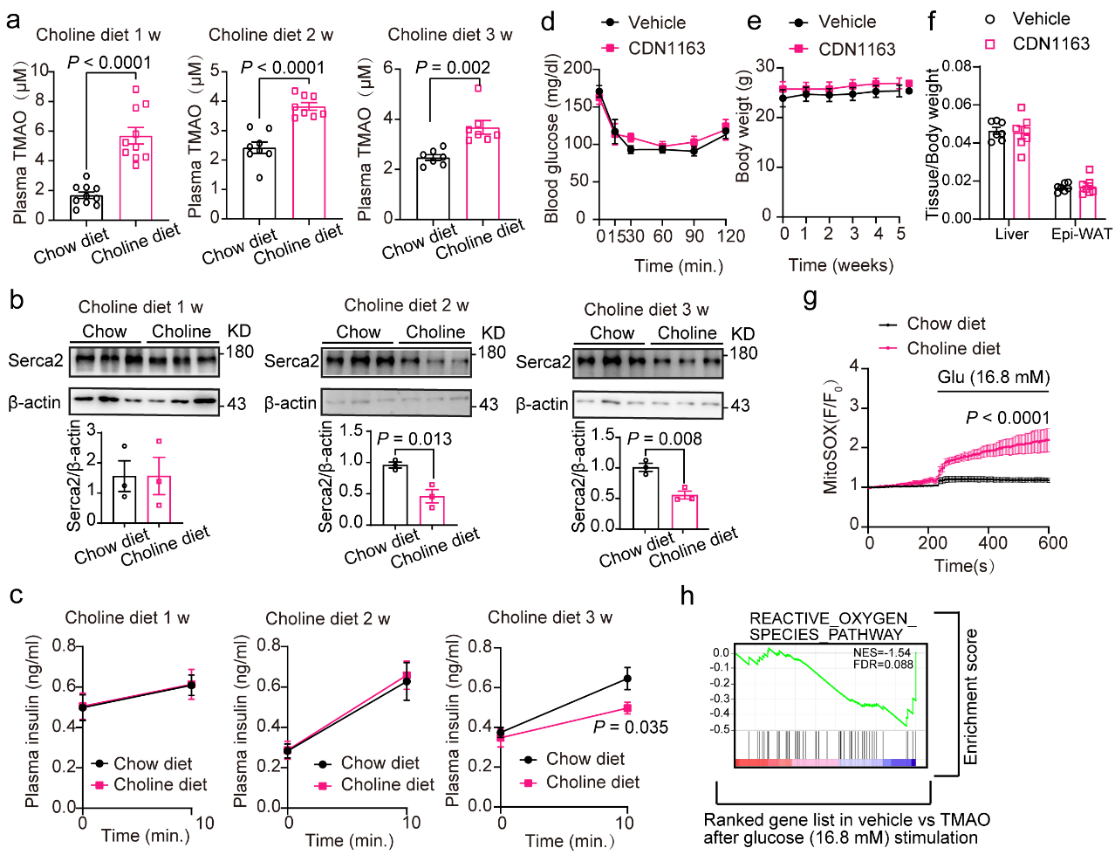

Supplementary Figure 5. TMAO enhanced the ROS pathway, related to Figure 5. **a** Plasma TMAO concentration in male chow diet- and choline diet-fed (1, 2, 3 weeks) mice. n=10 mice (1 week), n=8 mice (2 weeks), n=7 mice (3 weeks). **b** Serca2 protein levels in islets isolated from male chow diet- and choline diet-fed (1, 2, 3 weeks) mice. n=3 mice. **c** Plasma insulin levels after intraperitoneal injection of glucose for 0 and 10 min in male chow diet- and choline diet-fed (1, 2, 3 weeks) mice. n=7 mice (1, 3 weeks), n=10 mice (2 weeks). **d** ITTs of vehicle- (n=6 mice) and CDN1163-treated (26 d) male choline diet-fed mice (n=7 mice). **e** Body weight of vehicle- (n=7 mice) and CDN1163-treated male choline diet-fed mice (n=6 mice). **f** The ratio of tissue weight to body weight in vehicle- and CDN1163- treated (45 d) male choline diet-fed mice (n=7 mice). **g** Mitochondrial ROS in normal β cells from chow- (n=9 cells from 6 samples) and choline diet-fed (6 weeks) mice (n=6 cells from 6 samples). **h** GSEA plot of the ROS pathway in vehicle- and TMAO (100 nM, 18 h)-treated MIN6 cells after high-glucose (16.8 mM) stimulation. n=3 biologically independent cell samples. Statistical significance was calculated (**a-c, g**) by two-sided Student's t-test. The data are presented

as mean ± SEM. Mice: C57BL/6J, choline diet feeding from 8 weeks old (**a-g**). Source data are provided as a Source Data file.

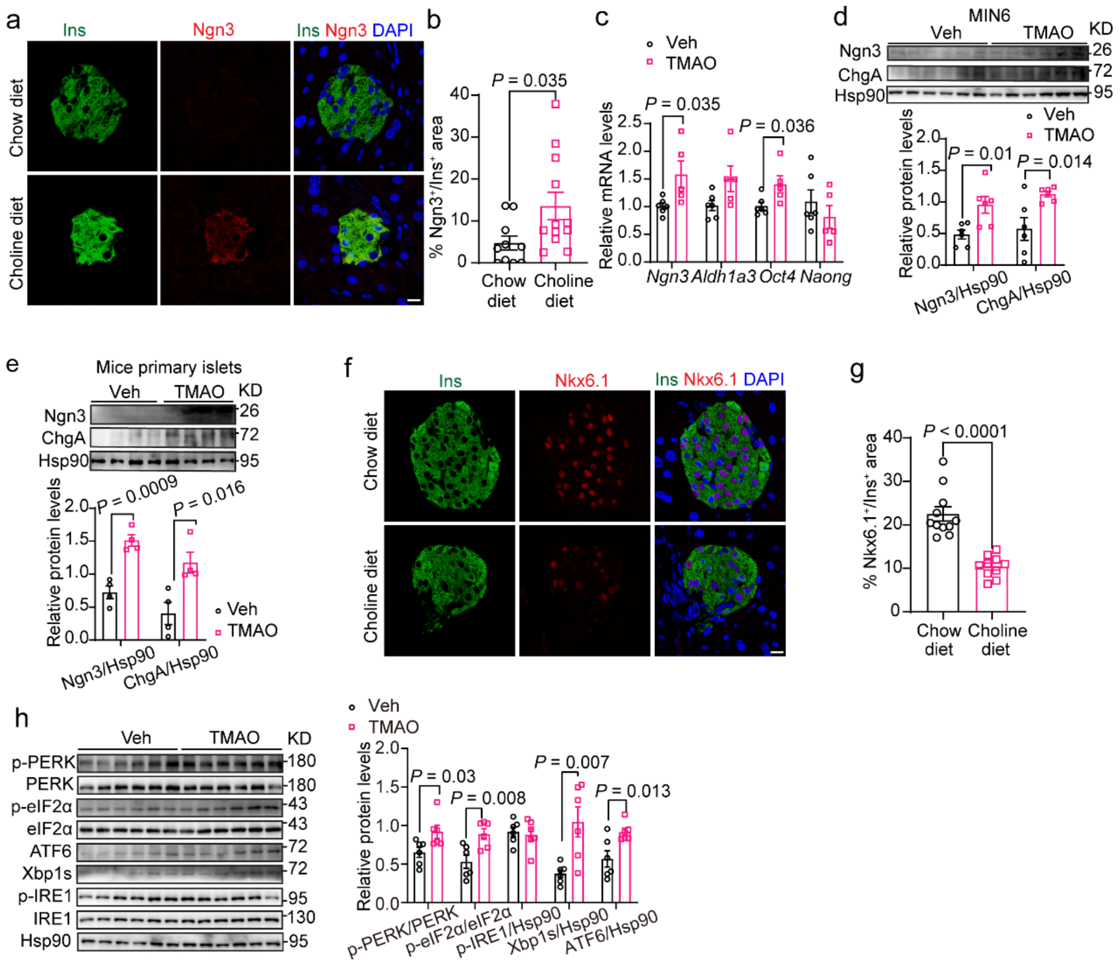

Supplementary Figure 6. TMAO increased dedifferentiation marker levels, related to Figure 6. a-b Immunofluorescence of insulin (green), Ngn3 (Neurogenin3, red) and DAPI (blue) in male chow- and choline diet-fed (13 weeks) mice (**a**). This step was followed by measurements of the % Ngn3$^+$/Ins$^+$ area (**b**). n=10 (chow), or 12 (choline). Scale bar, 10 μm. **c** Dedifferentiation marker mRNA levels in MIN6 cells treated with and without TMAO (100 nM, 9 d). n=6 (vehicle), or 5 (TMAO) biologically independent cell samples. **d** Ngn3 and ChgA protein levels in MIN6 cells treated with and without TMAO (100 nM, 9 d). n=6 biologically independent cell samples. **e** Ngn3 and ChgA protein levels in male mouse primary islets treated with and without TMAO (100 nM, 9 d). n=4 biologically independent cell samples. **f-g**

Immunofluorescence of insulin (green), Nkx6.1 (red) and DAPI (blue) in male chow- and choline diet-fed (13 weeks) mice (**f**). This step was followed by measurements of % Nkx6.1^{+}/Ins^{+} area (**g**). n=11 (chow), or 10 (choline). Scale bar, 10 μm. **h** ER stress-related protein levels in MIN6 cells treated with or without TMAO (100 nM, 9 d). n=6 biologically independent cell samples. Statistical significance was calculated (**b-e, g-h**) by two-sided Student's t-test. The data are presented as mean ± SEM. Mice: C57BL/6J, choline diet feeding from 8 weeks old (**a-b, f-g**); C57BL/6J, 15 weeks old (**e**). Source data are provided as a Source Data file.

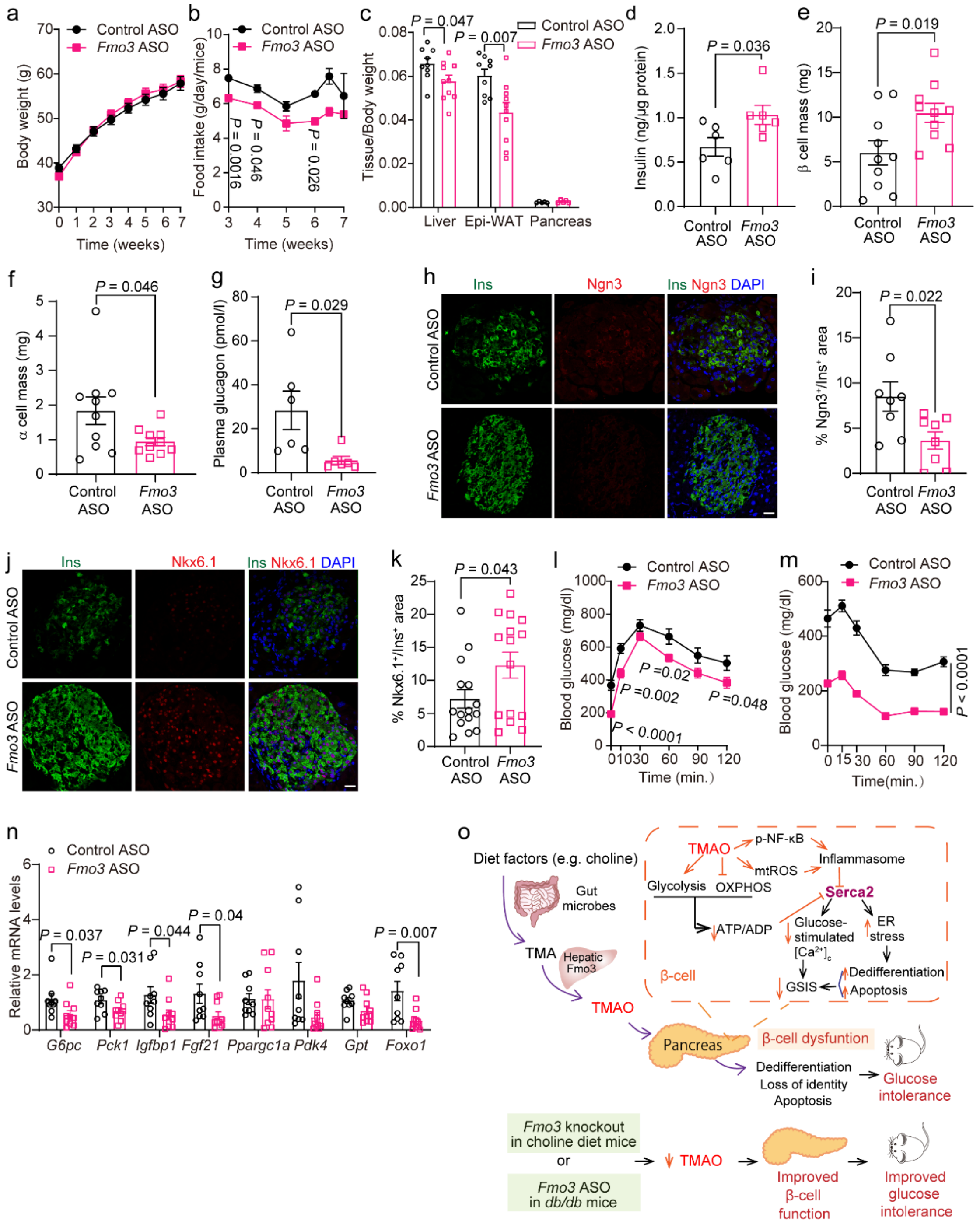

Supplementary Figure 7. Deficiency of Fmo3 improved insulin sensitivity in *db/db* mice, related to Figure 7. a Body weight during ASO treatment in *db/db* mice. n=10 mice. **b** Food intake during ASO treatment in *db/db* mice. n=2. **c** Tissue weight to body weight ratio of ASO-treated *db/db* mice. n=9 (control ASO), or 10 (*Fmo3* ASO) mice except for pancreas weight to body weight ratio of ASO-treated *db/db* mice were n=5. **d** KCl (30 mM) stimulated insulin secretion in primary islets from 5 weeks of ASO

treatment in male *db/db* mice. n=6 biologically independent islet samples. **e-f** Quantification of β- and α- cell mass in male control and *Fmo3* ASO-treated (10 weeks) *db/db* mice. n=10 mice. **g** Plasma glucagon levels in male control and *Fmo3* ASO-treated (6 weeks) *db/db* mice. n=6 mice. **h-i** Immunofluorescence for insulin (green), Ngn3 (red) and DAPI (blue) in male control and *Fmo3* ASO-treated *db/db* mice (**h**). This step was followed by measurements of the % $Ngn3^{+}/Ins^{+}$ area (**i**). n=8 from 6 mice. Scale bar, 20 μm. **j-k** Immunofluorescence for insulin (green), Nkx6.1 (red) and DAPI (blue) in male control and *Fmo3* ASO-treated *db/db* mice (**j**). Then, the % $Nkx6.1^{+}/Ins^{+}$ area was measured (**k**). n=15 from 6 mice. Scale bar, 20 μm. (**l**) IPGTTs after 3 weeks of ASO treatment in male *db/db* mice. n=10 mice. **m** ITTs after 7 weeks of ASO treatment in male *db/db* mice. n=8 (control ASO), or 9 (*Fmo3* ASO) mice. **n** Hepatic gluconeogenesis-related mRNA levels in ASO-treated male *db/db* mice. n=9 (control ASO), or 10 (*Fmo3* ASO) mice. **o** Graphic summary on TMAO impairs β-cell function and glucose tolerance through Serca2. The gut microbes metabolize diet factors such as choline to form TMA and hepatic Fmo3 oxidizes TMA to TMAO. Plasma TMAO levels are elevated in both diabetic mice and human subjects. TMAO inhibits calcium transients through NLRP3 inflammasome-related cytokines and induced Serca2 loss. Long-term TMAO exposure promotes β-cell ER stress, dedifferentiation, and apoptosis. Consequently, the β-cell function and glucose tolerance are impaired. Inhibition of TMAO production through either genetic knockdown or antisense oligomers of *Fmo3*, the TMAO-producing enzyme, improves β-cell and glucose tolerance in both *db/db* and choline diet-fed mice. Statistical significance was calculated (**b-g, i, k-n**) by two-sided Student's t-test. The data are presented as mean ± SEM. Mice, *db/db*, ASO treatment (50 mg/kg body weight) from 6 weeks old (**a-n**). Source data are provided as a Source Data file.

Supplementary Tables

Supplementary Table 1. Information on control subjects who provided liver samples

Group	Sex	Age
Control	Female	20-30
	Male	30-40
	Male	60-70

Supplementary Table 2. Information on NAFLD subjects who provided liver samples

Group	Sex	Age	BMI	HbA1c (%)	Insulin (pM)	TG (mM)	TC (mM)
NAFLD	Female	50-60	25.5	5.5	53.8	1.76	6.26
	Male	40-50	19.8	5.7	142.0	0.57	3.5
	Female	50-60	26.7	5.8	NA	1.13	2.42
	Male	60-70	22.7	6.6	445.4	0.83	3.82
	Male	60-70	31.4	5.0	339.9	2.41	6.25
	Male	50-60	47.4	5.2	124.2	2.53	2.90
	Female	60-70	44.4	6.2	167.8	0.90	5.34
	Male	60-70	44.6	6.1	213.4	1.21	5.28
	Female	60-70	40.0	6.8	159.6	3.71	4.2
	Male	50-60	31.8	10	50.3	1.33	4.58

NA: not available

Supplementary Table 3. Information on donors who provided islets

Group	Age	Sex	BMI	HbA1c
Normal	50-60	Male	25.95	NA
Normal	40-50	Male	29.05	5.3
Normal	50-60	Female	36.73	5.8
Normal	40-50	Male	20.05	5.3

NA: not available

Supplementary Table 4. Composition of diets

Diets	Carbohydrate (% calories)	Protein (% calories)	Fat (% calories)	Choline (%)
0.08% choline diet	68%	21%	12%	0.08
1% choline diet	68%	21%	12%	1
