# Supplementary material for: Trimethylamine N-oxide impairs β-cell function and glucose tolerance
Source: Nat Commun. 2024 Mar 21;15:2526. doi: 10.1038/s41467-024-46829-0 (PMC10957989; doi:10.1038/s41467-024-46829-0)
Supplement: Supplementary file 3 — Description of Additional Supplementary Files [file 41467_2024_46829_MOESM3_ESM.pdf]

Description of Additional Supplementary Files

Supplementary Data 1: Information on donors who provided serum

Supplementary Data 2: Oligos used in this study

Supplementary Data 3: Key resources table
