# Supplementary material for: Trimethylamine N-oxide impairs β-cell function and glucose tolerance
Source: Nat Commun. 2024 Mar 21;15:2526. doi: 10.1038/s41467-024-46829-0 (PMC10957989; doi:10.1038/s41467-024-46829-0)
Supplement: Supplementary file 4 — Supplementary Data 1 [file 41467_2024_46829_MOESM4_ESM.docx]

**Supplementary Data 1. Information on donors who provided serum**

| Group | Sex | Age | Fasting glucose (mM) | Fasting insulin (uIU/ml) | Diagnostic |
| --- | --- | --- | --- | --- | --- |
| Control | Female | 30-40 | 4.4 | 8.96 | Hyperthyrea |
|  | Male | 30-40 | 4.9 | 7.39 | Myasthenia gravis |
|  | Female | 20-30 | 5.2 | 5.87 | Irregular menses |
|  | Female | 20-30 | 4.8 | 15.09 | Irregular menses |
|  | Female | 30-40 | 5.3 | 8.57 | Irregular menses |
|  | Male | 10-20 | 4.7 | 14.13 | Polycystic ovary syndrome |
|  | Male | 30-40 | 5.7 | 11.21 | Debilitation |
|  | Female | 30-40 | 6.7 | 9.05 | Secondary menostasis |
|  | Female | 30-40 | 5.3 | 14.36 | Irregular menses |
|  | Female | 20-30 | 5.2 | 11.71 | Irregular menses |
|  | Female | 80-90 | 9.9 | 13.07 | Dizzy |
|  | Female | 20-30 | 5.5 | 3.23 | Irregular menses |
|  | Female | 30-40 | 5.4 | 12.82 | Irregular menses |
|  | Female | 0-10 | 4.8 | 9.46 | Pubertas examine |
|  | Male | 40-50 | 5 | 7.38 | Kidney calculi |
|  | Female | 60-70 | 5.3 | 13.72 | Rheumatoid arthritis |
|  | Male | 10-20 | 5.1 | 11.54 | Hypopituitarism |
|  | Female | 20-30 | 4 | 5.29 | Pregnancy |
|  | Female | 20-30 | 5.8 | 13.29 | Primary menostasis |
|  | Female | 30-40 | 5.3 | 9.99 | Irregular menses |
|  | Male | 40-50 | 5.1 | 12.15 | Thyroid function examination |
|  | Female | 20-30 | 4.6 | 11.78 | Pregnancy |
|  | Female | 10-20 | 4.1 | 7.55 | Irregular menses |
|  | Female | 30-40 | 4.3 | 7.2 | Natural abortion |
|  | Female | 30-40 | 5.2 | 7.26 | Irregular menses |
|  | Female | 30-40 | 5.4 | 5.65 | Irregular menses |
|  | Male | 30-40 | 4.7 | 14.88 | Kidney calculi |
|  | Female | 0-10 | 5.5 | 9.23 | Pubertas examine |
|  | Female | 10-20 | 4.6 | 12.15 | Hypogonadotropic hypogonadism |
|  | Female | 20-30 | 5.7 | 6.1 | Irregular menses |
|  | Female | 30-40 | 5.3 | 18.5 | Irregular menses |
|  | Female | 20-30 | 5.4 | 8.31 | Irregular menses |
|  | Female | 20-30 | 5.8 | 11.54 | Polycystic ovary syndrome |
|  | Female | 60-70 | 6 | 7.23 | Thyroid nodule |
|  | Female | 20-30 | 4.8 | 8.97 | Irregular menses |
|  | Female | 20-30 | 5.3 | 6.54 | Irregular menses |
|  | Female | 20-30 | 4.4 | 7.98 | Irregular menses |
|  | Female | 10-30 | 5.1 | 8.87 | Hirsutism examination |
|  | Female | 50-60 | 7.7 | 7.49 | Hypercortisolism |
|  | Female | 30-40 | 5 | 12.79 | Pregnancy |
|  | Female | 30-40 | 5 | 10.23 | Irregular menses |
|  | Male | 10-20 | 4.8 | 4.8 | Pubertas examine |
|  | Female | 20-30 | 4.9 | 10.4 | Irregular menses |
|  | Male | 50-60 | 5.7 | 9.23 | Hypopotassemia |
|  | Female | 40-50 | 5.8 | 11.91 | Numbness |
|  | Female | 20-30 | 4.6 | 9.01 | Irregular menses |
|  | Female | 30-40 | 5 | 5.24 | Natural abortion |
|  | Female | 20-30 | 4.5 | 12.46 | Irregular menses |
|  | Male | 30-40 | 5 | 9.77 | Myasthenia gravis |
|  | Female | 20-30 | 5 | 12.81 | Irregular menses |
|  | Female | 60-70 | 6.1 | 12.81 | Kidney calculi |
|  | Female | 60-70 | 6 | 7.23 | Thyroid nodule |
|  | Female | 70-80 | 5.7 | 4.85 | Thyroid nodule |
|  | Female | 30-40 | 4.5 | 5.33 | Irregular menses |
|  | Female | 40-50 | 4.9 | 4.15 | Kidney calculi |
|  | Female | 40-50 | 5.1 | 12.06 | Debilitation |
|  | Female | 0-10 | 5 | 2.14 | Pubertas examine |
|  | Male | 40-50 | 4.5 | 7.99 | Myasthenia gravis |
|  | Male | 10-20 | 5.5 | 5.25 | Pubertas examine |
|  | Female | 30-40 | 5.4 | 8.85 | Irregular menses |
| T2D | Female | 40-50 | 9.8 | 14.81 | Type 2 Diabetes |
|  | Male | 60-70 | 6.5 | 3.96 | Type 2 Diabetes |
|  | Female | 70-80 | 7.3 | 37.69 | Type 2 Diabetes |
|  | Male | 40-50 | 13 | 8.95 | Type 2 Diabetes |
|  | Female | 50-60 | 17.2 | 5.91 | Type 2 Diabetes |
|  | Female | 40-50 | 11.4 | 5.9 | Type 2 Diabetes |
|  | Male | 60-70 | 8.6 | 10.95 | Type 2 Diabetes |
|  | Male | 50-60 | 22.7 | 2.26 | Type 2 Diabetes |
|  | Female | 60-70 | 8.3 | 27.16 | Type 2 Diabetes |
|  | Female | 70-80 | 11 | 11.93 | Type 2 Diabetes |
|  | Male | 60-70 | 8.9 | 14.62 | Type 2 Diabetes |
|  | Male | 40-50 | 13.5 | 4.12 | Type 2 Diabetes |
|  | Male | 50-60 | 6.7 | 10.32 | Type 2 Diabetes |
|  | Male | 60-70 | 9.6 | 6.8 | Type 2 Diabetes |
|  | Male | 60-70 | 9.9 | 12.61 | Type 2 Diabetes |
|  | Female | 60-70 | 6 | 7.6 | Type 2 Diabetes |
|  | Female | 50-60 | 6.2 | 17.72 | Type 2 Diabetes |
|  | Female | 60-70 | 16.1 | 5.31 | Type 2 Diabetes |
|  | Female | 60-70 | 14.1 | 11.22 | Type 2 Diabetes |
|  | Female | 60-70 | 10.8 | 8.84 | Type 2 Diabetes |
|  | Male | 50-60 | 10.9 | 13.23 | Type 2 Diabetes |
|  | Female | 80-90 | 9.6 | 8.95 | Type 2 Diabetes |
|  | Female | 70-80 | 11.6 | 5.88 | Type 2 Diabetes |
|  | Male | 50-60 | 8.4 | 7.85 | Type 2 Diabetes |
|  | Female | 69-70 | 7.2 | 25.79 | Type 2 Diabetes |
|  | Male | 50-60 | 8.1 | 20.92 | Type 2 Diabetes |
|  | Male | 30-40 | 6.2 | 14.72 | Type 2 Diabetes |
|  | Male | 50-60 | 8 | 67.75 | Type 2 Diabetes |
|  | Male | 30-40 | 6.2 | 14.72 | Type 2 Diabetes |
|  | Male | 50-60 | 8 | 67.75 | Type 2 Diabetes |
|  | Female | 40-50 | 6 | 10.01 | Type 2 Diabetes |
|  | Male | 20-30 | 12.4 | 30.24 | Type 2 Diabetes |
|  | Male | 50-60 | 11.5 | 4.34 | Type 2 Diabetes |
|  | Female | 50-60 | 5.3 | 40.42 | Type 2 Diabetes |
|  | Female | 50-60 | 7.2 | 10.27 | Type 2 Diabetes |
|  | Male | 50-60 | 8 | 17.83 | Type 2 Diabetes |
|  | Male | 40-50 | 8.1 | 19.21 | Type 2 Diabetes |
|  | Female | 40-50 | 17.1 | 5.99 | Type 2 Diabetes |
|  | Female | 60-70 | 15.2 | 6.43 | Type 2 Diabetes |
|  | Male | 40-50 | 10.9 | 12.58 | Type 2 Diabetes |
|  | Female | 20-30 | 6.3 | 19.4 | Type 2 Diabetes |
|  | Male | 30-40 | 13.1 | 15.56 | Type 2 Diabetes |
|  | Male | 60-70 | 8.8 | 16.09 | Type 2 Diabetes |
|  | Male | 60-70 | 6.9 | 9.64 | Type 2 Diabetes |
|  | Male | 70-80 | 5.9 | 19.97 | Type 2 Diabetes |
|  | Female | 40-50 | 10.2 | 10.44 | Type 2 Diabetes |
|  | Male | 70-80 | 9.5 | 23.01 | Type 2 Diabetes |
|  | Male | 40-50 | 18.8 | 7.63 | Type 2 Diabetes |
|  | Female | 60-70 | 8 | 16.67 | Type 2 Diabetes |
|  | Male | 30-40 | 14 | 11.51 | Type 2 Diabetes |
|  | Female | 60-70 | 8.5 | 14.68 | Type 2 Diabetes |
|  | Female | 30-40 | 13.8 | 6.13 | Type 2 Diabetes |
|  | Male | 50-60 | 8.2 | 12.5 | Type 2 Diabetes |
|  | Male | 80-90 | 9.6 | 29.35 | Type 2 Diabetes |
|  | Male | 50-60 | 6.1 | 13.44 | Type 2 Diabetes |
|  | Male | 50-60 | 6.6 | 13.01 | Type 2 Diabetes |
|  | Female | 50-60 | 15.8 | 6.85 | Type 2 Diabetes |
|  | Female | 60-70 | 11.8 | 19.2 | Type 2 Diabetes |
|  | Female | 40-50 | 8.8 | 17.42 | Type 2 Diabetes |
|  | Female | 70-80 | 10.3 | 22.65 | Type 2 Diabetes |
