# Supplementary material for: Trimethylamine N-oxide impairs β-cell function and glucose tolerance
Source: Nat Commun. 2024 Mar 21;15:2526. doi: 10.1038/s41467-024-46829-0 (PMC10957989; doi:10.1038/s41467-024-46829-0)
Supplement: Supplementary file 5 — Supplementary Data 2 [file 41467_2024_46829_MOESM5_ESM.docx]

**Supplementary Data 2. Oligos used in this study**

| Oligo name | Sequence (5'-3') | Purpose |
| --- | --- | --- |
| Mouse *Fmo3* | Forward ACTGGTGGTACACAAGGCAG | qPCR |
| Mouse *Fmo3* | Reverse ATGGTCCCATCCTCAAACACA | qPCR |
| Human *FMO3* | Forward CAATTTACCGACAGCCATCTCT | qPCR |
| Human *FMO3* | Reverse ACGTTAGGCTTTACGGACACA | qPCR |
| Mouse *Serca1* | Forward TGTTTGTCCTATTTCGGGGTG | qPCR |
| Mouse *Serca1* | Reverse AATCCGCACAAGCAGGTCTTC | qPCR |
| Mouse *Serca2 all* | Forward TCGACAGGACAGAAAGAGTGTG | qPCR |
| Mouse *Serca2 all* | Reverse AAACTGAATTCAACTCACCAGC | qPCR |
| Mouse *Serca2a* | Forward GCTCATTTTCCAGATCACACCG | qPCR |
| Mouse *Serca2a* | Reverse GTTACTCCAGTATTGCGGGTTG | qPCR |
| Mouse *Serca2b* | Forward ACCTTTGCCGCTCATTTTCCAG | qPCR |
| Mouse *Serca2b* | Reverse AGGCTGCACACACTCTTTACC | qPCR |
| Mouse *Serca3* | Forward GGAGCAGTTTGAGGACCTCTT | qPCR |
| Mouse *Serca3* | Reverse GGCCACGAGAATTAGCATGATG | qPCR |
| Mouse *Tnf* | Forward CCCTCACACTCAGATCATCTTCT | qPCR |
| Mouse *Tnf* | Reverse GCTACGACGTGGGCTACAG | qPCR |
| Mouse *Nos2* | Forward GTTCTCAGCCCAACAATACAAGA | qPCR |
| Mouse *Nos2* | Reverse GTGGACGGGTCGATGTCAC | qPCR |
| Mouse *Il6* | Forward TAGTCCTTCCTACCCCAATTTCC | qPCR |
| Mouse *Il6* | Reverse TTGGTCCTTAGCCACTCCTTC | qPCR |
| Mouse *Il1β* | Forward GCAACTGTTCCTGAACTCAACT | qPCR |
| Mouse *Il1β* | Reverse ATCTTTTGGGGTCCGTCAACT | qPCR |
| Mouse *Cxcl1* | Forward CTGGGATTCACCTCAAGAACATC | qPCR |
| Mouse *Cxcl1* | Reverse CAGGGTCAAGGCAAGCCTC | qPCR |
| Mouse *Adgre1* | Forward CTTTGGCTATGGGCTTCCAGTC | qPCR |
| Mouse Adgre1 | Reverse GCAAGGAGGACAGAGTTTATCGTG | qPCR |
| Mouse *Ccl2* | Forward TTAAAAACCTGGATCGGAACCAA | qPCR |
| Mouse *Ccl2* | Reverse GCATTAGCTTCAGATTTACGGGT | qPCR |
| Mouse *Foxp3* | Forward CCCATCCCCAGGAGTCTTG | qPCR |
| Mouse *Foxp3* | Reverse ACCATGACTAGGGGCACTGTA | qPCR |
| Mouse *Tnfβ* | Forward CCACCTCTTGAGGGTGCTTG | qPCR |
| Mouse *Tnfβ* | Reverse CATGTCGGAGAAAGGCACGAT | qPCR |
| Mouse *Rorγt* | Forward GACCCACACCTCACAAATTGA | qPCR |
| Mouse *Rorγt* | Reverse AGTAGGCCACATTACACTGCT | qPCR |
| Mouse *Itgax* | Forward CTGGATAGCCTTTCTTCTGCTG | qPCR |
| Mouse *Itgax* | Reverse GCACACTGTGTCCGAACTCA | qPCR |
| Mouse *Sox9* | Forward CGGAACAGACTCACATCTCTCC | qPCR |
| Mouse *Sox9* | Reverse GCTTGCACGTCGGTTTTGG | qPCR |
| Mouse *Ngn3* | Forward CCAAGAGCGAGTTGGCACT | qPCR |
| Mouse *Ngn3* | Reverse CGGGCCATAGAAGCTGTGG | qPCR |
| Mouse *Aldh1a3* | Forward GGGTCACACTGGAGCTAGGA | qPCR |
| Mouse *Aldh1a3* | Reverse CTGGCCTCTTCTTGGCGAA | qPCR |
| Mouse *Oct4* | Forward CACCATCTGTCGCTTCGAGG | qPCR |
| Mouse *Oct4* | Reverse CACCATCTGTCGCTTCGAGG | qPCR |
| Mouse *Nanog* | Forward TCTTCCTGGTCCCCACAGTTT | qPCR |
| Mouse *Nanog* | Reverse GCAAGAATAGTTCTCGGGATGAA | qPCR |
| Mouse *Ins1* | Forward CACTTCCTACCCCTGCTGG | qPCR |
| Mouse *Ins1* | Reverse ACCACAAAGATGCTGTTTGACA | qPCR |
| Mouse *Ins2* | Forward GCTTCTTCTACACACCCATGTC | qPCR |
| Mouse *Ins2* | Reverse AGCACTGATCTACAATGCCAC | qPCR |
| Mouse *Iapp* | Forward CCACTTGAGAGCTACACCTGT | qPCR |
| Mouse *Iapp* | Reverse GAACCAAAAAGTTTGCCAGGC | qPCR |
| Mouse *Pdx1* | Forward CCCCAGTTTACAAGCTCGCT | qPCR |
| Mouse *Pdx1* | Reverse CTCGGTTCCATTCGGGAAAGG | qPCR |
| Mouse *Nkx6.1* | Forward CTGCACAGTATGGCCGAGATG | qPCR |
| Mouse *Nkx6.1* | Reverse CCGGGTTATGTGAGCCCAA | qPCR |
| Mouse *Mafa* | Forward AGGAGGAGGTCATCCGACTG | qPCR |
| Mouse *Mafa* | Reverse CTTCTCGCTCTCCAGAATGTG | qPCR |
| Mouse *Ucn3* | Forward AAGCCTCTCCCACAAGTTCTA | qPCR |
| Mouse *Ucn3* | Reverse GAGGTGCGTTTGGTTGTCATC | qPCR |
| Mouse *Pcsk1* | Forward CTTTCGCCTTCTTTTGCGTTT | qPCR |
| Mouse *Pcsk1* | Reverse TCCGCCGCCCATTCATTAAC | qPCR |
| Mouse *Gck* | Forward TGAGCCGGATGCAGAAGGA | qPCR |
| Mouse *Gck* | Reverse GCAACATCTTTACACTGGCCT | qPCR |
| Mouse *Neurod1* | Forward ATGACCAAATCATACAGCGAGAG | qPCR |
| Mouse *Neurod1* | Reverse TCTGCCTCGTGTTCCTCGT | qPCR |
| Mouse *Mnx1* | Forward TCTATGGACACCCGGTCTACA | qPCR |
| Mouse *Mnx1* | Reverse CCCCAAGAGGTTCGACTGC | qPCR |
| Mouse *Foxo1* | Forward CCCAGGCCGGAGTTTAACC | qPCR |
| Mouse *Foxo1* | Reverse GTTGCTCATAAAGTCGGTGCT | qPCR |
| Mouse *G6pc* | Forward CGACTCGCTATCTCCAAGTGA | qPCR |
| Mouse *G6pc* | Reverse GTTGAACCAGTCTCCGACCA | qPCR |
| Mouse *Pck1* | Forward CTGCATAACGGTCTGGACTTC | qPCR |
| Mouse *Pck1* | Reverse CAGCAACTGCCCGTACTCC | qPCR |
| Mouse *Igfbp1* | Forward ATCAGCCCATCCTGTGGAAC | qPCR |
| Mouse *Igfbp1* | Reverse TGCAGCTAATCTCTCTAGCACTT | qPCR |
| Mouse *Fgf21* | Forward CTGCTGGGGGTCTACCAAG | qPCR |
| Mouse *Fgf21* | Reverse CTGCGCCTACCACTGTTCC | qPCR |
| Mouse *Ppargc1a* | Forward TATGGAGTGACATAGAGTGTGCT | qPCR |
| Mouse *Ppargc1a* | Reverse CCACTTCAATCCACCCAGAAAG | qPCR |
| Mouse *Pdk4* | Forward AGGGAGGTCGAGCTGTTCTC | qPCR |
| Mouse *Pdk4* | Reverse GGAGTGTTCACTAAGCGGTCA | qPCR |
| Mouse *Gpt* | Forward TCCAGGCTTCAAGGAATGGAC | qPCR |
| Mouse *Gpt* | Reverse CAAGGCACGTTGCACGATG | qPCR |
| Control ASO | CCTTCCCTGAAGGTTCCTCC | ASO |
| *Fmo3* ASO | TGGAAGC ATTTGCCTTTAAA | ASO |
| *Fmo3* sgRNA | TGGGAAAGTCATCGGGATAGGGG | sgRNA |
