# Supplementary material for: Trimethylamine N-oxide impairs β-cell function and glucose tolerance
Source: Nat Commun. 2024 Mar 21;15:2526. doi: 10.1038/s41467-024-46829-0 (PMC10957989; doi:10.1038/s41467-024-46829-0)
Supplement: Supplementary file 6 — Supplementary Data 3 [file 41467_2024_46829_MOESM6_ESM.docx]

**Supplementary Data 3. Key resources table**

| REAGENT or RESOURCE | SOURCE | IDENTIFIER |
| --- | --- | --- |
| Antibodies |  |  |
| Recombinant Anti-FMO3 antibody | Abcam | Cat. Ab126711 |
| HSP90 Monoclonal Antibody | Proteintech | Cat. 60318-1-Ig |
| ATP2A2/SERCA2 (D51B11) Rabbit mAb | Cell Signaling Technology | Cat. 9580 |
| Beta Actin Monoclonal Antibody | Proteintech | Cat. 66009-1-Ig |
| Phospho-PERK (Thr980) (16F8) Rabbit mAb | Cell Signaling Technology | Cat. 3179 |
| PERK (C33E10) Rabbit mAb | Cell Signaling Technology | Cat. 3192 |
| Phospho-eIF2α (Ser51) (D9G8) XP® Rabbit mAb | Cell Signaling Technology | Cat. 3398 |
| eIF2α (D7D3) XP® Rabbit mAb | Cell Signaling Technology | Cat. 5324 |
| Recombinant Anti-IRE1 (phospho S724) antibody  IRE1α (14C10) Rabbit mAb | Abcam  Cell Signaling Technology | Cat. ab124945  Cat. 3294 |
| XBP-1s (E9V3E) Rabbit mAb | Cell Signaling Technology | Cat. 40435 |
| Anti-ATF6 antibody | Abcam | Cat. ab203119 |
| Anti-Sox9 antibody | Sigma-Aldrich | Cat. AB5535 |
| Anti-Pdx1 antibody | Abcam | Cat. ab219207 |
| Anti-Nkx6.1 antibody | Abcam | Cat. ab221549 |
| Anti-Neurogenin3/Ngn3 | Abcam | Cat. ab176124 |
| Anti-Chromograin A/ChgA | Abcam | Cat. ab254322 |
| Cleaved Caspase-3 (Asp175) (5A1E) Rabbit mAb | Cell Signaling Technology | Cat. 9664 |
| Caspase-3 (D3R6Y) Rabbit mAb | Cell Signaling Technology | Cat.14220 |
| Cleaved PARP (Asp214) (7C9) Mouse mAb (Mouse Specific) | Cell Signaling Technology | Cat. 9548 |
| PARP (46D11) Rabbit mAb | Cell Signaling Technology | Cat. 9532 |
| NF-κB p65 (D14E12) XP® Rabbit mAb | Cell Signaling Technology | Cat. 8242 |
| Phospho-NF-κB p65 (Ser536) (93H1) Rabbit mAb | Cell Signaling Technology | Cat. 3033 |
| Mouse Reactive Inflammasome Antibody Sampler Kit | Cell Signaling Technology | Cat. 20836 |
| Rabbit Anti-Phospho-PPAR-γ (Ser273) antibody | Bioss | Cat. bs-4888R |
| PPAR-γ (C26H12) Rabbit mAb | Cell Signaling Technology | Cat. 2435 |
| Human/Mouse/Bovine Insulin Antibody | RD | Cat. 182410 |
| Recombinant Anti-Glucagon antibody  Rabbit Polyclonal ACE2 Antibody [Biotin]  CD24 Antibody-Biotin | Abcam  Novus  Miltenyi Biotec | Cat. ab92517  Cat. NBP1-76614B  Cat. 130-101-982 |
| Chemicals |  |  |
| TMAO | Sigma | Cat. 317594 |
| D9-TMAO | Cambridge Isotopes | Cat. DML-4779-1 |
| [6]-gingerol | Sigma | Cat. 345868 |
| CDN1163 | Sigma | Cat. SML1682 |
| MCC950 sodium | MCE | Cat. HY-12815A |
| Glucose | Sinopharm Chemical Reagent Co., Ltd. | Cat. 10010592 |
| GLP-1 (7-36) | MCE | Cat. HY-P0054 |
| L-Arginine monohydrochloride | Sigma | Cat. A5131 |
| Fatty acid free bovine serum albumin | Equitech-Bio, Inc. | Cat. BAH66 |
| Collagenase from Clostridium histolytium | Sigma | Cat. C9263 |
| Human recombinant insulin | Lilly | Cat. HI0219 |
| Fura-2, AM | Invitrogen | Cat. F1221 |
| mitoSOX Red, Molecular Probes  phosphoenolpyruvate  ATP disodium salt  pyruvate kinase  lactate dehydrogenase  NADH  digitonin  protease inhibitor cocktail  PMSF  4-Bromo A23187  Anti-Biotin MicroBeads | Invitrogen  Sigma  Sigma  Sigma  Sigma  Sigma  Sigma  Sigma  Roche  MCE  Miltenyi Biotec | Cat. M36008  Cat. P7127  Cat. A3377  Cat. P1903-1KU  Cat. 59747  Cat. N8129  Cat. D141  Cat. P8340  Cat. 10837091001  Cat. HY-N6694  Cat. 130-090-485 |
| Critical Commercial Assays |  |  |
| Mouse Ultrasensitive Insulin ELISA Jumbo | ALPCO | Cat. 80-INSMSU-E-10 |
| Mouse C-peptide ELISA | ALPCO | Cat. 80-CPTMS-E01 |
| Mercodia Insulin ELISA | Mercodia | Cat. 10-1113-01 |
| ATP Colorimetric/Fluorometric Assay Kit | BioVision | Cat. K354-100 |
| ADP/ATP Ratio Assay Kit | Abcam | Cat. Ab65313 |
| Cell Counting Kit-8 | DOJINDO | Cat. CK17 |
| Seahorse XF Cell Mito Stress Test Kit | Seahorse | Cat. 103015-100 |
| Seahorse XF glycolysis stress Test Kit | Seahorse | Cat. 103020-100 |
| Glucose Uptake-Glo Assay | Promega | Cat. J1342 |
| Cell lines |  |  |
| MIN6  αTC1-6 | Institute of Biophysics, Chinese Academy of Sciences  ATCC | N.A.  CRL-2934 |
| Islets |  |  |
| Mice primary islets | This paper | N.A. |
| Human primary islets | Tianjin First central hospital | N.A. |
| Animals |  |  |
| Mouse: C57BL/6J for choline diet | Vital Rive | N.A. |
| Mouse: C57BL/6J *Fmo3^-/-^* | Biocytogen | N.A. |
| Mouse: C57BL/6J *Fmo3^+/+^* | Biocytogen | N.A. |
| Mouse: *db/db* | GemPharmatech | N.A. |
| Oligonucleotides |  |  |
| Control ASO | Eurogentec | Cat. 2298097 |
| *Fmo3* ASO | Eurogentec | Cat. 2298098 |
| Software and Algorithms |  |  |
| ImageJ | NIH | <https://imagej.nih.gov/ij/> |
| Prism | GraphPad | <https://www.graphpad.com> |
| QuantStudio Design & Analysis Software v1.4.3 | Thermo | <https://www.thermofisher.cn/cn/zh/home.html> |
| Other |  |  |
| Chow diet  0.08% choline diet | Beijing HFK  Bioscience  MoIDiets | Cat.1035  M18072501 |
| 1% choline diet | MoIDiets | M18072502 |
| 60% HFD | Research Diets | Cat.D12492 |
