# Supplementary material for: Trimethylamine N-oxide impairs β-cell function and glucose tolerance
Source: Nat Commun. 2024 Mar 21;15:2526. doi: 10.1038/s41467-024-46829-0 (PMC10957989; doi:10.1038/s41467-024-46829-0)
Supplement: Supplementary file 7 — Reporting Summary [file 41467_2024_46829_MOESM7_ESM.pdf]

nature portfolio

Corresponding author(s): Pingping Li

Last updated by author(s): Feb 23, 2024

# Reporting Summary

Nature Portfolio wishes to improve the reproducibility of the work that we publish. This form provides structure for consistency and transparency in reporting. For further information on Nature Portfolio policies, see our Editorial Policies and the Editorial Policy Checklist.

## Statistics

For all statistical analyses, confirm that the following items are present in the figure legend, table legend, main text, or Methods section.

| n/a | Confirmed | |
|---|---|---|
| ☐ | ☒ | The exact sample size ($n$) for each experimental group/condition, given as a discrete number and unit of measurement |
| ☐ | ☒ | A statement on whether measurements were taken from distinct samples or whether the same sample was measured repeatedly |
| ☐ | ☒ | The statistical test(s) used AND whether they are one- or two-sided<br>*Only common tests should be described solely by name; describe more complex techniques in the Methods section.* |
| ☒ | ☐ | A description of all covariates tested |
| ☒ | ☐ | A description of any assumptions or corrections, such as tests of normality and adjustment for multiple comparisons |
| ☐ | ☒ | A full description of the statistical parameters including central tendency (e.g. means) or other basic estimates (e.g. regression coefficient) AND variation (e.g. standard deviation) or associated estimates of uncertainty (e.g. confidence intervals) |
| ☐ | ☒ | For null hypothesis testing, the test statistic (e.g. $F$, $t$, $r$) with confidence intervals, effect sizes, degrees of freedom and $P$ value noted<br>*Give P values as exact values whenever suitable.* |
| ☒ | ☐ | For Bayesian analysis, information on the choice of priors and Markov chain Monte Carlo settings |
| ☒ | ☐ | For hierarchical and complex designs, identification of the appropriate level for tests and full reporting of outcomes |
| ☒ | ☐ | Estimates of effect sizes (e.g. Cohen's $d$, Pearson's $r$), indicating how they were calculated |

*Our web collection on statistics for biologists contains articles on many of the points above.*

## Software and code

Policy information about availability of computer code

| | |
|---|---|
| Data collection | Standard software and the respective analysis tools provided by manufacturers were listed in the methods (Zeiss microsystems, QuantStudio 3, Excel 2016, Image J 2.0.0, etc.). No software was used other than that listed in the Methods. |
| Data analysis | Student's t-test analysis were done by using Excel 2016 and Prism GraphPad 8.0. All microscopy data was collected and analyzed using the Zeiss microsystems and ZEN software. Immunofluorescence and Western blots images analysis was performed by Image J 2.0.0. |

For manuscripts utilizing custom algorithms or software that are central to the research but not yet described in published literature, software must be made available to editors and reviewers. We strongly encourage code deposition in a community repository (e.g. GitHub). See the Nature Portfolio guidelines for submitting code & software for further information.

## Data

Policy information about availability of data

All manuscripts must include a data availability statement. This statement should provide the following information, where applicable:

- Accession codes, unique identifiers, or web links for publicly available datasets
- A description of any restrictions on data availability
- For clinical datasets or third party data, please ensure that the statement adheres to our policy

The RNA sequencing data generated in this study have been deposited in the Gene Expression Omnibus database under accession code GSE243083 [https://www.ncbi.nlm.nih.gov/geo/query/acc.cgi?acc=GSE243083]. All other data generated or analyzed during this study are included in this article (and its supplementary information files). Source data are provided with this paper.

## Research involving human participants, their data, or biological material

Policy information about studies with human participants or human data. See also policy information about sex, gender (identity/presentation), and sexual orientation and race, ethnicity and racism.

| | |
|---|---|
| Reporting on sex and gender | Human primary islets were provided by Tianjin First Center Hospital (China), male=3, female=1. Human livers were provided by Wenzhou Medical University (Zhejiang, China), for NAFLD model, male=6, female=4, for the control, male=2, female=1. Human serum was provided by Peking Union Medical College Hospital, for T2D group, male=32, female=28, for the control, male=12, female=48. |
| Reporting on race, ethnicity, or other socially relevant groupings | All participants are Asians in this study. |
| Population characteristics | The specific informations of donors for providing livers, islets and serum were provided in Supplementary Tables 1-3 and Supplementary Data 1. |
| Recruitment | The samples were received anonymously. |
| Ethics oversight | All protocols for livers were approved by the First Affiliated Medical Hospital of Wenzhou Medical University (no. 2016-246) , All protocols for islets were approved by Ethical Committee of Tianjin First Central Hospital (no. 2016N086KY). All protocols for serum were approved by Ethical Committee of Peking Union Medical College Hospital (no. ZS-1274). |

Note that full information on the approval of the study protocol must also be provided in the manuscript.

# Field-specific reporting

Please select the one below that is the best fit for your research. If you are not sure, read the appropriate sections before making your selection.

[x] Life sciences [ ] Behavioural & social sciences [ ] Ecological, evolutionary & environmental sciences

For a reference copy of the document with all sections, see nature.com/documents/nr-reporting-summary-flat.pdf

# Life sciences study design

All studies must disclose on these points even when the disclosure is negative.

| | |
|---|---|
| Sample size | No statistical method was used to determine animal's sample size. Sample size was chosen based on experience with the used experimental models in the field of cell biology and animal experiments. Detailed description of the statistical methods used for the analyse, appears in the paper. At least three biological replicates were achieved for experiments in cells, such sample sizes are typical for the in vitro experiments. For in vivo experiments, a sample size of n≥5 mice was used per experimental group. |
| Data exclusions | No data was excluded in this study. |
| Replication | Replication attempts were successful. The experiments were repeated with at least three biological and/or technical replicates to ensure reproducibility. The detail was indicated in the Methods section. |
| Randomization | For animal studies, db/db mice were grouped according to bodyweight, fasting blood glucose before they were assigned into different treatment groups. For all other experiments, mice or samples were randomly allocated into group and then earmarked by an independent researcher. |
| Blinding | All experiments were performed in a non-blinded manner. Investigators were not blinded as endpoint criteria was defined prior to experiment. |

# Reporting for specific materials, systems and methods

We require information from authors about some types of materials, experimental systems and methods used in many studies. Here, indicate whether each material, system or method listed is relevant to your study. If you are not sure if a list item applies to your research, read the appropriate section before selecting a response.

## Materials & experimental systems

| n/a | Involved in the study | |
|---|---|---|
| | x | Antibodies |
| | x | Eukaryotic cell lines |
| x | | Palaeontology and archaeology |
| | x | Animals and other organisms |
| x | | Clinical data |
| x | | Dual use research of concern |
| x | | Plants |

## Methods

| n/a | Involved in the study | |
|---|---|---|
| x | | ChIP-seq |
| x | | Flow cytometry |
| x | | MRI-based neuroimaging |

# Antibodies

Antibodies used

Western Blots: anti-Fmo3 (Abcam, clone EPR6968, cat. #Ab126711, 1:500), anti-Hsp90 (Proteintech, clone 3F11C1, cat. #60318-1-lg, 1:5000), anti-ATP2A2/SERCA2(Cell Signaling Technology (CST), clone D51B11, cat. #9580, 1:1000), anti-β Actin (Proteintech, clone 2D4H5, cat. #66009-l-lg, 1:5000), anti-phospho-PERK(Thr980)(CST, clone 16F8,  cat. #3179, 1:1000), anti-PERK(CST, clone C33E10, cat. #3192, 1:1000), anti-phospho eIF2α (Ser51)(CST, clone D9G8, cat. #3398, 1:1000), anti-eIF2α (CST, clone D7D3, cat. #5324, 1:1000), anti-phospho-IRE1 (Ser724) (Abcam, clone EPR5253, cat. #ab124945, 1:500), anti-IRE1 (CST, clone 14C10, cat. #3294, 1:1000), anti-XBP-1s (CST, clone E9V3E, cat. #40435, 1:1000), anti-ATF6 (Abcam, cat. #ab203119, 1:500), anti-Sox9 (Sigma Aldrich, cat. #AB5535, 1:500),anti-Pdx1 (Abcam, clone EPR22002, cat. #ab219207, 1:1000), anti-Nkx6.1 (Abcam, clone EPR20405, cat. #ab221549, 1:1000), anti-Neurogenin3 (Abcam, cat. #ab176124, 1:500), anti-ChromograinA (Abcam, clone EPR22537-248, cat. #ab254322, 1:1000),anti-cleaved caspase-3 (Asp175)(CST, clone 5A1E, cat. #9664, 1:1000), anti-caspase-3 (CST, clone D3R6Y, cat. #14220, 1:1000), anti-cleaved PARP (Asp214)(CST, clone 7C9, cat. #9548 1:1000), anti-PARP (CST, clone 46D11, cat. #9532, 1:1000), anti-NF-κB p65 (CST, clone D14E12, cat. #8242, 1:1000), anti-phospho-NF-κB p65 (Ser536)(CST, clone 93H1, cat. #3033, 1:1000), anti-NLRP3 (CST, clone D4D8T, cat. #15101, 1:1000), anti-ASC (CST, clone D2W8U, cat. #67824, 1:1000), anti-cleaved caspase-1 (CST, clone E2G2I, cat. #89332, 1:1000), anti-cleaved IL-1β (CST, clone E7V2A, cat. #63124, 1:1000), anti- IL-1β (CST, clone D6D6T, cat. #31202, 1:1000), anti-AIM2 (CST, cat. #63660, 1:1000), anti-phospho-PPAR-γ (ser273)(Bioss, cat. #bs-4888R, 1:500), anti-PPAR-γ (CST, clone C26H12, cat. #2435, 1:1000). HRP-conjugated goat anti-rabbit (Jackson ImmunoResearch, cat. #111-035-003, 1:10,000), HRP-conjugated goat anti-mouse (Jackson ImmunoResearch, cat. #115-035-003, 1:10,000)
Immunofluorescence: anti-insulin (R&D Systems, Clone #182410, Cat #MAB1417, 1:200), anti-glucagon (Abcam, clone EP3070, #ab92517, 1:200), anti-Sox9 (Sigma Aldrich, #AB5535, 1:500),anti-Pdx1 (Abcam, clone EPR22002, cat. #ab219107, 1:1000), anti-Nkx6.1 (Abcam, clone EPR20405, cat. #ab221594, 1:100), anti-Neurogenin3 (Abcam, cat. #ab176124, 1:200), anti-cleaved caspase-3 (Asp175)(CST, clone 5A1E, cat. #9664, 1:400), Alexa Fluor 488 goat anti-rat (Invitrogen, cat. #A11006, 1:200), Alexa Fluor 546 goat anti-rabbit (Invitrogen, cat. #A11035, 1:200), Alexa Fluor 647 goat anti-mouse (Invitrogen, cat. #A32728, 1:200).
Magnetic Activated Cell Sorting: anti-ACE2-Biotin (Novus, cat. #NBP1-76614B, 1:10),anti-CD24-Biotin (Miltenyi Biotec, clone M1/69, cat. #130-101-982, 1:10)

Validation

Validation statements for all used antibodies are available at the websites of the commercial providers.
anti-Fmo3 (Abcam, clone EPR6968, cat. #Ab126711), https://www.abcam.com/products/primary-antibodies/fmo3-antibody-epr6968-ab126711.html, Liu A  et al, 2022.
anti-Hsp90 (Proteintech, clone 3F11C1, cat. #60318-1-Ig), https://www.ptgcn.com/products/HSP90-Antibody-60318-1-Ig.htm, Liu XF et al, 2022.
anti-ATP2A2/SERCA2(CST, clone D51B11, cat. #9580), https://www.cellsignal.cn/products/primary-antibodies/atp2a2-serca2-d51b11-rabbit-mab/9580, Ha Thu Nguyen, et al, 2023.
anti-β Actin (Proteintech, clone 2D4H5, cat. #66009-l-lg), https://www.ptgcn.com/products/Pan-Actin-Antibody-66009-1-Ig.htm, Duanfang Cao, et al, 2023.
anti-phospho-PERK(Thr980)(CST, clone 16F8,  cat. #3179), https://www.cellsignal.cn/products/primary-antibodies/phospho-perk-thr980-16f8-rabbit-mab/3179, Riyaz Mohamed, et al, 2023.
anti-PERK(CST, clone C33E10, cat. #3192), https://www.cellsignal.cn/products/primary-antibodies/perk-c33e10-rabbit-mab/3192, Riyaz Mohamed, et al, 2023.
anti-phospho eIF2α (CST, clone D9G8, cat. #3398), https://www.cellsignal.cn/products/primary-antibodies/phospho-eif2a-ser51-d9g8-xp-rabbit-mab/3398, Kuang-Chi Lai, et al, 2024.
anti-eIF2α (CST, clone D7D3, cat. #5324), https://www.cellsignal.cn/products/primary-antibodies/eif2a-d7d3-xp-174-rabbit-mab/5324, Hung-Chieh Lee, et al, 2023.
anti-phospho-IRE1 (Ser724) (Abcam, clone EPR5253, cat. #ab124945), https://www.abcam.com/products/primary-antibodies/ire1-phospho-s724-antibody-epr5253-ab124945.html, Chung AW  et al, 2022.
anti-IRE1 (CST, clone 14C10, cat. #3294), https://www.cellsignal.cn/products/primary-antibodies/ire1a-14c10-rabbit-mab/3294, Elif Ertürk, et al, 2023.
anti-XBP-1s (CST, clone E9V3E, cat. #40435), https://www.cellsignal.cn/products/primary-antibodies/xbp-1s-e9v3e-rabbit-mab/40435, Huahua Wang, et al, 2023.
anti-ATF6 (Abcam, #ab203119), https://www.abcam.com/products/primary-antibodies/atf6-antibody-ab203119.html, Yu R,  et al, 2022.
anti-Sox9 (Sigma Aldrich, #AB5535), https://www.sigmaaldrich.cn/CN/zh/product/mm/ab5535, Oeztuerk-Winder, F., et al, 2012.
anti-Pdx1 (Abcam, clone EPR22002, cat. #ab219207), https://www.abcam.com/products/primary-antibodies/lgr5-antibody-ab219207.html, Chang ZY, et al, 2022.
anti-Nkx6.1 (Abcam, clone EPR20405, cat. #ab221549), https://www.abcam.com/products/primary-antibodies/nkx61-antibody-

epr20405-ab221549.html, Zhang X, et al, 2022.
anti-Neurogenin3 (Abcam, #ab176124), https://www.abcam.com/products/primary-antibodies/neurogenin3ngn-3-antibody-ab176124.html, Gao D, et al, 2022.
anti-ChromograinA (Abcam, EPR22537-248, #ab254322), https://www.abcam.com/products/primary-antibodies/chromogranin-a-antibody-epr22537-248-ab254322.html.
anti-cleaved caspase-3 (Asp175)(CST, 5A1E, #9664), https://www.cellsignal.cn/products/primary-antibodies/cleaved-caspase-3-asp175-5a1e-rabbit-mab/9664, Sujin Choi, et al, 2024.
anti-caspase-3 (CST, D3R6Y, #14220), https://www.cellsignal.cn/products/primary-antibodies/caspase-3-d3r6y-rabbit-mab/14220, Elif Ertürk, et al, 2023.
anti-cleaved PARP (Asp214)(CST, 7C9, #9548), https://www.cellsignal.cn/products/primary-antibodies/cleaved-parp-asp214-7c9-mouse-mab/9548, Carly S Wilder, et al, 2023.
anti-PARP (CST, 46D11, #9532), https://www.cellsignal.cn/products/primary-antibodies/parp-46d11-rabbit-mab/9532, Kuang-Chi Lai, et al, 2024.
anti-NF-κB p65 (CST, D14E12, #8242), https://www.cellsignal.cn/products/primary-antibodies/nf-kb-p65-d14e12-xp-174-rabbit-mab/8242, Xiaoqian Guo, et al, 2024.
anti-phospho-NF-κB p65 (Ser536)(CST, 93H1, #3033), https://www.cellsignal.cn/products/primary-antibodies/phospho-nf-kb-p65-ser536-93h1-rabbit-mab/3033, Jian Ge, et al, 2023.
anti-NLRP3 (CST, D4D8T, #15101), https://www.cellsignal.cn/products/primary-antibodies/nlrp3-d4d8t-rabbit-mab/15101, Dong Zhang, et al, 2024.
anti-ASC (CST, D2W8U, #67824), https://www.cellsignal.cn/products/primary-antibodies/asc-tms1-d2w8u-rabbit-mab/67824, Lun Li, et al, 2023.
anti-cleaved caspase-1 (Asp296)(CST, E2G2I, #89332), https://www.cellsignal.cn/products/primary-antibodies/cleaved-caspase-1-asp296-e2g2i-rabbit-mab/89332, Mingxiang Ding, et al, 2024.
anti-cleaved IL-1β (Asp117)(CST, E7V2A, #63124), https://www.cellsignal.cn/products/primary-antibodies/cleaved-il-1b-asp117-e7v2a-rabbit-mab/63124, Huachun Cui, et al, 2023.
anti- IL-1β (CST, D6D6T, #31202), https://www.cellsignal.cn/products/primary-antibodies/il-1b-d6d6t-rabbit-mab/31202, Pin-Yi Liu, et al, 2023.
anti-AIM2 (CST, #63660), https://www.cellsignal.cn/products/primary-antibodies/aim2-antibody/63660, Vanessa Delcroix, et al, 2023.
anti-phospho-PPAR-γ (ser273)(Bioss, #bs-4888R), http://bioss.com.cn/prolook_03.asp?id=AF08169606015370&pro37=1, Zhang Yudian, et al, 2023.
anti-PPAR-γ (CST, C26H12, #2435), https://www.cellsignal.cn/products/primary-antibodies/pparg-c26h12-rabbit-mab/2435, Mimi Zhang, et al, 2023.
HRP-conjugated goat anti-rabbit (Jackson ImmunoResearch, cat. #111-035-003), https://www.jacksonimmuno.com/catalog/products/111-035-003.
HRP-conjugated goat anti-mouse (Jackson ImmunoResearch, cat. #115-035-003), https://www.jacksonimmuno.com/catalog/products/115-035-003.
anti-insulin (R&D Systems, Clone #182410, Cat #MAB1417), https://www.rndsystems.com/cn/products/human-mouse-bovine-insulin-antibody-182410_mab1417, Y Wakabayash, et al, 2022.
anti-glucagon (Abcam, EP3070, #ab92517), https://www.abcam.com/products/primary-antibodies/glucagon-antibody-ep3070-ab92517.html, Benderradji H, et al, 2022.
Alexa Fluor 488 goat anti-rat (Invitrogen, cat. #A11006), https://www.thermofisher.cn/cn/zh/antibody/product/Goat-anti-Rat-IgG-H-L-Cross-Adsorbed-Secondary-Antibody-Polyclonal/A-11006.
Alexa Fluor 546 goat anti-rabbit (Invitrogen, cat. #A11035), https://www.thermofisher.cn/cn/zh/antibody/product/Goat-anti-Rabbit-IgG-H-L-Highly-Cross-Adsorbed-Secondary-Antibody-Polyclonal/A-11035.
Alexa Fluor 647 goat anti-mouse (Invitrogen, cat. #A32728), https://www.thermofisher.cn/cn/zh/antibody/product/Goat-anti-Mouse-IgG-H-L-Highly-Cross-Adsorbed-Secondary-Antibody-Polyclonal/A32728.
anti-ACE2-Biotin (Novus, #NBP1-76614B), https://www.novusbio.com/products/ace-2-antibody_nbp1-76614b.
anti-CD24-Biotin (Miltenyi Biotec, M1/69, #130-101-982), https://www.miltenyibiotec.com/CN-en/products/cd24-antibody-anti-mouse-m1-69.html#conjugate=biotin:size=30-ug-in-1-ml.

# Eukaryotic cell lines

Policy information about cell lines and Sex and Gender in Research

| | |
|---|---|
| Cell line source(s) | MIN6 cells were obtained from Dr. Tao Xu, a professor from the Institute of Biophysics, Chinese Academy of Sciences. αTC1-6 cells were purchased from ATCC (Catalog: CRL-2934).The mice primary islets were obtained from males. The human primary islets were obtained from males and females. |
| Authentication | MIN6 and αTC1-6 cells were authenticated by the cell morphology and function. Primary islets were authenticated by staining with dithizone (DTZ). |
| Mycoplasma contamination | Cell lines were routinely tested for potential mycoplasma contamination by using commercial mycoplasma detection kits (Londa, LT07-418). The detection assays are selective biochemical tests to detect mycoplasma contamination in cell cultures. The kits exploit the activity of mycoplasmal enzymes which are found in the vast majority of about 200 mycoplasma species, but are not present in eukaryotic cells. All tests were negative. |
| Commonly misidentified lines (See ICLAC register) | There were no commonly misidentified lines in our study. |

## Animals and other research organisms

Policy information about studies involving animals; ARRIVE guidelines recommended for reporting animal research, and Sex and Gender in Research

| | |
|---|---|
| Laboratory animals | 6-8 weeks old C57BL/6J mice (Vital River Lab Animal Technology, Beijing, China), 6-8 weeks old db/db mice (Model Animal Research Center of Nanjing University), Fmo3 knockout mice (generated by Beijing Biocytogen, China). 16 weeks old Ins1-GCaMP6f mice (gifted by Prof. Liangyi Chen at Peking University). Male mice were hosted for all experiments. Mice were maintained in a temperature- (21-23°C) and humidity- (50-60%) controlled environment with a 12 h light/dark cycle (7 AM–7 PM). |
| Wild animals | No wild animals were used in the study. |
| Reporting on sex | It is much easier to induce disease in male mice. So male mice were hosted for disease model in our experiments. |
| Field-collected samples | No field collected samples were used in the study. |
| Ethics oversight | All experiments using animals were performed in accordance with protocols approved by the Animal Experimentation Ethics Committee of the Chinese Academy of Medical Sciences, and all procedures were conducted in accordance with the guidelines of the Institutional Animal Care and Use Committees of the Chinese Academy of Medical Sciences. All animal procedures were consistent with the ARRIVE guidelines. |

Note that full information on the approval of the study protocol must also be provided in the manuscript.

## Plants

| | |
|---|---|
| Seed stocks | No plants were involved in the study. |
| Novel plant genotypes | No plants were involved in the study. |
| Authentication | No plants were involved in the study. |
